# Supplementary material for: Ablation of prostaglandin E2 signalling through dual receptor knockout in CAR T cells enhances therapeutic efficacy in solid tumours
Source: Nat Biomed Eng. 2026 Feb 11;10(4):697–710. doi: 10.1038/s41551-025-01610-6 (PMC13099425; doi:10.1038/s41551-025-01610-6)
Supplement: Supplementary file 1 — Reporting Summary [file 41551_2025_1610_MOESM1_ESM.pdf]

nature portfolio

Corresponding author(s): Prof. Sebastian Kobold

Last updated by author(s): Nov 10, 2025

Reporting Summary

Nature Portfolio wishes to improve the reproducibility of the work that we publish. This form provides structure for consistency and transparency in reporting. For further information on Nature Portfolio policies, see our Editorial Policies and the Editorial Policy Checklist.

Statistics

For all statistical analyses, confirm that the following items are present in the figure legend, table legend, main text, or Methods section.

n/a	Confirmed	
☐	☒	The exact sample size (*n*) for each experimental group/condition, given as a discrete number and unit of measurement
☐	☒	A statement on whether measurements were taken from distinct samples or whether the same sample was measured repeatedly
☐	☒	The statistical test(s) used AND whether they are one- or two-sided<br>*Only common tests should be described solely by name; describe more complex techniques in the Methods section.*
☒	☐	A description of all covariates tested
☐	☒	A description of any assumptions or corrections, such as tests of normality and adjustment for multiple comparisons
☐	☒	A full description of the statistical parameters including central tendency (e.g. means) or other basic estimates (e.g. regression coefficient) AND variation (e.g. standard deviation) or associated estimates of uncertainty (e.g. confidence intervals)
☐	☒	For null hypothesis testing, the test statistic (e.g. *F*, *t*, *r*) with confidence intervals, effect sizes, degrees of freedom and *P* value noted<br>*Give P values as exact values whenever suitable.*
☒	☐	For Bayesian analysis, information on the choice of priors and Markov chain Monte Carlo settings
☒	☐	For hierarchical and complex designs, identification of the appropriate level for tests and full reporting of outcomes
☒	☐	Estimates of effect sizes (e.g. Cohen's *d*, Pearson's *r*), indicating how they were calculated

Our web collection on statistics for biologists contains articles on many of the points above.

Software and code

Policy information about availability of computer code

Data collection	LRSFortessa Cell Analyzer and FACS Canto II Flow Cytometer - BD FACSDiva (BD Biosciences)<br>CytoFLEX LX, - CytExpert (Beckmann Coulter)<br>xCELLigence RTCA MP&SP - RTCA Software Pro Agilent<br>Berthold Tristar 3 - MikroWin<br>IVIS Lumina X5, Perkin Elmer - Living Image 4.4., Perkin Elmer<br>MiSeq Illunina, Illumina<br>Eclipse NiE fluorescent microscope, Nikon
Data analysis	Excel (Microsoft Office Professional Plus 2016), FlowJo (BD Biosciences), Prism (Graphpad), CytExpert v(Beckmann Coulter), RTCA Software Pro (Agilent), AffinityDesigner (Serif), IVIS Lumina X5, Perkin Elmer - Living Image 4.4., Perkin Elmer, NIS-Elements AR software (Nikon)

For manuscripts utilizing custom algorithms or software that are central to the research but not yet described in published literature, software must be made available to editors and reviewers. We strongly encourage code deposition in a community repository (e.g. GitHub). See the Nature Portfolio guidelines for submitting code & software for further information.

Data

Policy information about availability of data

All manuscripts must include a data availability statement. This statement should provide the following information, where applicable:

- Accession codes, unique identifiers, or web links for publicly available datasets
- A description of any restrictions on data availability
- For clinical datasets or third party data, please ensure that the statement adheres to our policy

The main data supporting the results in this study are available within the paper and its extended data. The raw datasets generated in this study are available on the Open Data LMU repository at https://data.ub.uni-muenchen.de/713/ in accordance with the principles of open science of the European Research Council (ERC). Whole Genome Sequencing data will be made available for research purposes from the corresponding authors on reasonable request and regulatory clearance.

Research involving human participants, their data, or biological material

Policy information about studies with human participants or human data. See also policy information about sex, gender (identity/presentation), and sexual orientation and race, ethnicity and racism.

Reporting on sex and gender	not applicable
Reporting on race, ethnicity, or other socially relevant groupings	not applicable
Population characteristics	not applicable
Recruitment	Patient tumor samples were collected during surgical procedures at the Dana-Farber/Harvard Cancer Center.
Ethics oversight	Patient tumor samples were collected and analyzed according to Dana-Farber/Harvard Cancer Center (DF/HCC) Institutional Review Board (IRB)-approved protocols.

Note that full information on the approval of the study protocol must also be provided in the manuscript.

Field-specific reporting

Please select the one below that is the best fit for your research. If you are not sure, read the appropriate sections before making your selection.

☒ Life sciences ☐ Behavioural & social sciences ☐ Ecological, evolutionary & environmental sciences

For a reference copy of the document with all sections, see nature.com/documents/nr-reporting-summary-flat.pdf

Life sciences study design

All studies must disclose on these points even when the disclosure is negative.

Sample size	Samples sizes were chosen based on previously run pilot experiments and are indicated in the figure legends as n values.
Data exclusions	All mice used in animal experiments were analyzed except for one mouse (Fig. 5 c-e), which had to be killed due to issues clearly not related to tumor burden or treatment (eye infection). Further, two spleens had to be excluded from Supplementary Figure 1 e Day 9 before data acquisition due to an error in sample preparation leading to cell loss.<br>In Fig. 5 c-e, an outlier test was run and outliers were excluded, as indicated in the figure legend.<br>For in vitro experiments, all technically sound repetitions with appropriate results in the positive and negative controls were included.
Replication	For each experiment, the number of independent repeats is clearly stated in the respective figure legends.<br>In vivo experiments have been performed once (Fig. 1 e-g), three time (Fig. 4a-d, Fig. 5a-b) or twice (Fig. 5c-f) , as indicated. Experiments involving patient-dervied tumor samples have been replicated once (Fig. 4g-i) and three times (Fig. 4f). All attempts at replication have been successful.<br>All in vitro experiments have been performed three times using T cells from different donors. All attempts at replication have been successful.
Randomization	Mice were stratified into treatment groups by averaging tumor size per group for all animal experiments.
Blinding	Researchers were blinded during data acquisition, but not analysis, of all animal experiments.

Reporting for specific materials, systems and methods

We require information from authors about some types of materials, experimental systems and methods used in many studies. Here, indicate whether each material, system or method listed is relevant to your study. If you are not sure if a list item applies to your research, read the appropriate section before selecting a response.

Materials & experimental systems

n/a	Involved in the study
☐	☒ Antibodies
☐	☒ Eukaryotic cell lines
☒	☐ Palaeontology and archaeology
☐	☒ Animals and other organisms
☒	☐ Clinical data
☒	☐ Dual use research of concern
☒	☐ Plants

Methods

n/a	Involved in the study
☒	☐ ChIP-seq
☐	☒ Flow cytometry
☒	☐ MRI-based neuroimaging

Antibodies

Antibodies used	Human Antibodies:<br>Fixable Viability Dye eFluor 780 (1:1000, Invitrogen, Cat.# 65-0865-14), APC anti-human CD3 (1:100, clone OKT3, Biolegend, Cat.# 317318), PE-Cy7 anti-human CD4 (1:100, OKT4, BioLegend, Cat.# 317414), BV605 anti-human CD8 (1:100, SK1, Biolegend, Cat.# 344742), BV421 anti-human CD69 (1:100, clone F50, BioLegend, Cat.#310930), PerCP-Cy5.5 anti-human CD25 (1:100, clone BC96, BioLegend, Cat.# 302626), FITC anti-human/mouse/rat c-myc (1:100, SH1-26E7.1.3, Miltenyi, Cat.# 130-116-485), AF647 anti-CREB (pS133)/ATF-1 (pS63) (1:20, clone J151-21 (RUO), BD Bioscience, Cat.# 558434).<br><br>Murine Antibodies:<br>Pacific Blue anti-CD4 (1:100, clone GK1.5, Biolegend, Cat.#: 100428), FITC anti-CD8 (1:100, clone 53-6.7, Biolegend, Cat.#: 100706), Pacific Blue anti-CD3 (1:100, clone 17A2, Biolegend, Cat.#: 100214), Alexa Fluor 700 anti-CD4 (1:100, clone GK1.5, Biolegend, Cat.#: 100430), BV785 anti-CD8 (1:100, clone 53-6.7, Biolegend, Cat.#: 100750), BV711 anti-CD45.1 (1:100, clone A20, Biolegend, Cat.#: 110739), APC anti-rat CD90/mouse CD90.1 (Thy-1.1) (1:100, clone OX-7, Biolegend, Cat.#: 202526), FITC anti-mouse CD4 (1:100, clone GK1.5, BioLegend, Cat.# 100406), Pacfic Blue anti-mouse CD8a (1:100, clone 53-6.7, BioLegend, Cat.# 100725), PE-Cy7 anti-mouse IFNgamma (1:100, clone XMG1.2, eBioscience, Cat.# 25-7311-82), anti-murine CD3 monoclonal Antibody (145-2C11) Functional Grade eBioscience™ (ThermoFisher Scientific, clone 145-2C11, catalog #16-0031-82), anti-murine CD28 monoclonal Antibody (CD28.2) Functional Grade eBioscience™ (ThermoFisher Scientific, clone 37.51, catalog #16-0281-82)
Validation	All antibodies have been validated by the manufacturer and documentation can be found on the respective websites.

Eukaryotic cell lines

Policy information about cell lines and Sex and Gender in Research

Cell line source(s)	Panc02 cells were generated as previously described (Karches and Benmebarek et al. Clin Cancer Res, 2019, Lesch and Blumenberg et al., Nat Biomed Eng, 2021)<br>D4M.3A-OVA cells were generated as previously described (Di Pilato et al., Nature 2019)<br>BxPC3 (from Max Schnurr, Munich, Germany)<br>Msto-hMSLN (from Max Schnurr, Munich, Germany)<br>293Vec-Eco (from Manuel Caruso, Quebec, Canada)<br>293Vec-RD114 (from Manuel Caruso, Quebec, Canada)
Authentication	STR DNA profiling of human cell lines.
Mycoplasma contamination	Cells were regularly tested for mycoplasma contamination using polymerase chain reaction (PCR). All cells used throughout this study were confirmed to be negative for mycoplasma contamination prior to their use.
Commonly misidentified lines<br>(See ICLAC register)	No commonly misidentified cell lines were used in the study.

Animals and other research organisms

Policy information about studies involving animals; ARRIVE guidelines recommended for reporting animal research, and Sex and Gender in Research

Laboratory animals	The following strains of Mus musculus were used:<br>WT mice: C57BL/6J (strain #000664)<br>OT-I mice: C57BL/6-Tg(TcraTcrb)1100Mjb/J (strain #003831)<br>CD45.1 mice: B6.SJL-Ptprca Pepcb/BoyJ (strain #002014)<br>CD90.1 mice: B6.PL-Thy1a/CyJ (strain #000406)<br>Ptger2−/− mice: B6.129-Ptger2tm1Brey/J (strain #004376)<br>Ptger4fl/fl mice : B6.129S6(D2)-Ptger4tm1.1Matb/BreyJ (strain #028102)<br>CD4Cre mice : B6.Cg-Tg(Cd4-cre)1Cwi/BfluJ (strain #022071)

	NXG mice: NXG (NOD-Prkdcscid-IL2rgTm1/Rj)<br><br>Mice were 6-38 weeks of age.
Wild animals	No wild animals were used in the study.
Reporting on sex	In all tumor models, sex of the mice was matched to sex of the transplanted tumor cell line (D4M.3A = male; BxPC3 = female, Msto = female).
Field-collected samples	No field collected samples were used in the study.
Ethics oversight	Local regulatory agency (Regierung von Oberbayern).

Note that full information on the approval of the study protocol must also be provided in the manuscript.

Plants

Seed stocks	not applicable
Novel plant genotypes	not applicable
Authentication	not applicable

Flow Cytometry

Plots

Confirm that:

☒ The axis labels state the marker and fluorochrome used (e.g. CD4-FITC).

☒ The axis scales are clearly visible. Include numbers along axes only for bottom left plot of group (a 'group' is an analysis of identical markers).

☒ All plots are contour plots with outliers or pseudocolor plots.

☒ A numerical value for number of cells or percentage (with statistics) is provided.

Methodology

Sample preparation	Tissue preparation:<br>- mechanical disintegration<br>- only for tumor tissue: collagense/DNase digestion (37°C, 30min)<br>- pass through a cell strainer to get single cell suspensions<br>- erythrocyte lysis if necessary<br>- surface staining (4°C, 30min)<br>- wash step with PBS<br>- FACS analysis in PBS<br><br>Cell culture experiments:<br>- wash T cells with PBS<br>- surface staining (4°C, 30min)<br>- wash with PBS<br>- if applicable: fixation and permeabilization for intracellular stainings<br>- intracellular stainings in Permeabilization Buffer<br>- FACS analysis
Instrument	LRSFortessa Cell Analyzer (BD Biosciences)<br>FACS Canto II Flow Cytometer (BD Biosciences)<br>CytoFLEX LX (Beckmann Coulter)
Software	BD FACSDiva (BD Biosciences)<br>CytExpert (Beckmann Coulter)<br>FlowJo (BD Biosciences)
Cell population abundance	No cell sorting was performed.
Gating strategy	T cell tracking in D4M3A-tumor bearing mice:

Gating strategy	FSC-A/SSC-A for exclusion of debris and dead cells, FCS-A/FSC-H to select single cells, fixable viability dye to gate on live cells, CD3+ cells to exclude non-T cells, CD45.1/CD90.1 to track respective cell populations<br><br>T cell tracking in BxPC3-tumor bearing mice:<br>FSC-A/SSC-A for exclusion of debris and dead cells, FCS-A/FSC-H to select single cells, fixable viability dye to gate on live cells, CD3+ cells to exclude non-T cells, cMyc and teLuc to track respective cell populations<br><br>in vitro CAR T cell experiments:<br>FSC-A/SSC-A for exclusion of debris and dead cells, FCS-A/FSC-H to select single cells, fixable viability dye to gate on live cells, then depending on the assay further gating on CD4/CD8/EdU/pCREB/IFNg/CD25/CD69

☐ Tick this box to confirm that a figure exemplifying the gating strategy is provided in the Supplementary Information.
